# Antimelanogenesis Effects of Leaf Extract and Phytochemicals from Ceylon Olive (*Elaeocarpus serratus*) in Zebrafish Model

**DOI:** 10.3390/pharmaceutics13071059

**Published:** 2021-07-10

**Authors:** Chi-Ya Huang, I-Hsuan Liu, Xiang-Zhe Huang, Hui-Jen Chen, Shang-Tzen Chang, Mei-Ling Chang, Yu-Tung Ho, Hui-Ting Chang

**Affiliations:** 1 School of Forestry and Resource Conservation, National Taiwan University, Taipei 106, Taiwan; r99625047@ntu.edu.tw (C.-Y.H.); r01625054@ntu.edu.tw (X.-Z.H.); r93625033@ntu.edu.tw (H.-J.C.); peter@ntu.edu.tw (S.-T.C.); b04605047@ntu.edu.tw (Y.-T.H.); 2 Department of Animal Science and Technology, National Taiwan University, Taipei 106, Taiwan; ihliu@ntu.edu.tw; 3 Department of Food Science, Nutrition, and Nutraceutical Biotechnology, Shih Chien University, Taipei 104, Taiwan; mlchang@g2.usc.edu.tw

**Keywords:** antimelanogenesis effect, *Elaeocarpus serratus*, melanin, tyrosinase inhibitor, zebrafish

## Abstract

The melanogenesis inhibition effect in zebrafish (*Danio rerio*) and antityrosinase activity of the ethanolic extract and its phytochemicals from Ceylon olive (*Elaeocarpus serratus* Linn.) leaves were investigated in this study. Among the leaf extract and four soluble fractions, the ethyl acetate soluble fraction exhibits the best antityrosinase and antimelanogenesis activities. One phenolic acid, gallic acid, and two flavonoids, myricetin and mearnsetin, are isolated from the active subfractions through the bioassay-guided isolation; their structures are elucidated based on the 1D and 2D NMR, FTIR, UV, and MS spectroscopic analyses. These compounds have significant antityrosinase activity whether using l-tyrosine or l-DOPA as the substrate; mearnsetin shows the optimal activity. In the enzyme kinetic investigation, both gallic acid and mearnsetin are the competitive-type inhibitors against mushroom tyrosinase, and myricetin acts as a mixed-type tyrosinase inhibitor. Leaf extract and an ethyl acetate soluble fraction show effective performance in the inhibition of melanin formation in zebrafish embryos. Mearnsetin also possesses a promising antimelanogenesis effect, which is superior to the positive control, arbutin. Results reveal that the Ceylon olive leaf extract and its phytochemicals, especially mearnsetin, have the potential to be used as antimelanogenesis and skin-whitening ingredients.

## 1. Introduction

Melanin plays an essential role in many biochemical functions; it provides protection for the skin from ultraviolet (UV) damage, the elimination of reactive oxygen species (ROS), and other biochemical reactions [1,2,3]. The excessive accumulation of melanin also brings about the hyperpigmentation, melasma, age spots, skin darkening, etc. [4,5]. In the eastern world, people are interesting in the issue of the whitening effect on the skin; the cosmetic industry is dedicated to developing skin-whitening cosmetics against melanogenesis. There are several mechanisms, including tyrosinase inhibitory activity, melanocyte removal, and interference with melanin synthesis to exhibit melanogenesis inhibitory activity. The promising reagents inhibiting melanogenesis have been investigated since the 1940s [2,4,6,7]. However, part melanogenesis inhibitors possess some side effects; e.g., hydroquinone may cause exogenous ochronosis and permanent hypomelanosis [5,8]. Excessive kojic acid may result in allergy and thyroid tumor [9,10].

Recently, researchers have been dedicated to finding promising melanogenic reagents with no/mild side effects from plant natural products [11,12,13]. Natural tyrosinase inhibitors from plant sources have been reported, including simple phenols, phenolic acids, stillbenes, flavonoids, lignans, terpenoids, quinoids, etc. [2,14,15,16]. Solano et al. stated that phenolic compounds and the other natural compounds have the great potential for melanin inhibition, e.g., arbutin (a phenol glucoside found in *Arctostaphylos uva-ursi*), the extract of green tea, alpha hydroxyl acid (AHA), ascorbic acid, and so on [5]. Arbutin is widely used as a key ingredient of commercial whitening products on the market [4,17]. Gallocatechin-3-*O*-gallate (GCG), epigallocatechin-3-*O*-gallate (EGCG), and epicatechin-3-*O*-gallate (ECG) were identified in the green tea extract; these compounds possess the good tyrosinase inhibitory efficacy [17]. Arctigenin, a lignan isolated from *Fructus arctii* extract, could reduce the melanin content of zebrafish (*Danio rerio*) embryo [18]. Lee et al. reported that the ginsenosides from *Panax ginseng* leaf and berry inhibit the melanogenesis of the zebrafish embryo; the active ginsenosides are ginsenoside Rh6, vina-ginsenoside R4, vina-ginsenoside R13, ginsenoside Rh23, and floralginsenoside A [19,20,21].

*Elaeocarpus serratus* Linn. (Tiliaceae), commonly called Ceylon olive, is distributed in Africa, Australia, and Southeast Asia. The pickle made of the edible fruit of Ceylon olive is common in Sri Lanka [22]. It is traditionally used to prevent head lice and dandruff in Sri Lanka. Bioactivities of *E. serratus* extract were evaluated in the related studies [22,23]. *E. serratus* leaf extract possessed antibacterial activity against *Plesiomonas*, *Salmonella typhi*, and *Proteus* spp. at the concentration of 400 μg/mL by the plate-hole diffusion assay [23]. *E. serratus* leaf extract had the potential of being an antitumor, antimicrobial, and pesticidal reagent in the cytotoxicity investigation of brine shrimp (*Artemia salina*) lethality assay; the LC_50_ of the leaf extract was 40 μg/mL against the brine shrimp [23]. Antioxidant flavonol glycosides, including myricitrin, mearnsetin 3-*O*-β-D-glucopyranoside, mearnsitrin, and tamarixetin 3-*O*-α-L-rhamnopyranoside, were isolated and identified from the *E. serratus* leaf extract [22].

The objectives of this study are to evaluate the effects of *E. serratus* leaf extract and its fractions on antityrosinase activity (in vitro) and melanogenesis inhibition activity in zebrafish (in vivo). Bioassay-guided fractionation and isolation techniques were carried out to find the promising compounds as potential antimelanogenesis ingredients. Developments of effective antimelanogenesis ingredients can increase the optimal utilization of plant natural products in pharmaceutical and cosmetic industries.

## 2. Materials and Methods

### 2.1. Plant Material and Extraction

Ceylon olive (*Elaeocarpus serratus*), around 50 years old, leaves were collected from National Taiwan University, Taipei, Taiwan in September. Fresh leaves (3.95 kg) were extracted with 95% ethanol for 7 days at room temperature. The filtered extract was dried under reduced pressure by a rotary evaporator with a bath temperature of 50 °C [24]. The yield of leaf extract (0.32 kg) was 12.0% (*w*/*w* of dried weight).

### 2.2. Liquid–Liquid Partition

The extract was subjected to successive organic solvent extraction in the increasing polarity order by liquid–liquid partition [24,25]. The leaf extract was fractionated into *n*-hexane soluble fraction (HF, 9.1%), ethyl acetate soluble fraction (EF, 11.3%), *n*-butanol soluble fraction (BF, 17.3%), and water soluble fraction (WF, 31.0%).

### 2.3. Column Chromatography and Thin Layer Chromatography

Bioactive ethyl acetate soluble fraction was further fractionated by silica gel column chromatography (CC). Open-column chromatography was carried out by stepwise elution with different ratio of *n*-hexane and ethyl acetate [26]. Nineteen subfractions (E1–E19) were obtained by thin-layer chromatography (TLC) [27]. Yields of subfractions were E1 (1.05%), E2 (8.21%), E3 (1.64%), E4 (0.74%), E5 (1.52%), E6 (0.86%), E7 (1.51%), E8 (9.50%), E9 (7.78%), E10 (3.97%), E11 (2.65%), E12 (8.49%), E13 (5.58%), E14 (4.43%), E15 (5.31%), E16 (1.14%), E17 (1.87%), E18 (0.76%), and E19 (2.96%).

### 2.4. High-Performance Liquid Chromatography

Active subfractions of ethyl acetate soluble fraction were analyzed and isolated by high-performance liquid chromatography (HPLC, L-2130, Hitachi, Tokyo, Japan) fitted with a preparative RP-18 column (Purospher^®^, STAR RP-18 end-capped, 250 × 10 mm, 5 μm). The gradient mobile phase consisted of water (A) and acetonitrile (B). The flow rate was 3 mL/min. The elution program involved a linear gradient from 30 to 35% B in A for 0–15 min, 35 to 100% B in A by 15–25 min, and followed by 5 min of equilibrium with 100% B. The eluted peaks were detected by UV detector at 254 nm [25,28].

### 2.5. Identification of Isolated Compounds

The structures of isolated compounds were determined and characterized by means of spectral analyses, including ultraviolet-visible spectroscopy (UV/VIS, V-550, Jasco, Tokyo, Japan), Fourier transform infrared spectroscopy (FTIR, FTS-40, Bio-rad, Hercules, CA, USA), and mass spectroscopy (MS, MAT-958, Finnigan, MA, USA). Nuclear magnetic resonance spectroscopy (NMR), including 1D (^1^H-NMR, 500 MHz; ^13^C-NMR, 125 MHz) and 2D NMR (HSQC and HMBC), were recorded with a Bruker AVIII NMR spectrometer (Bruker Avance, Rheinstetten, Germany) [29,30,31,32].

### 2.6. Antityrosinase Assay and Enzyme Kinetic Study

In vitro antityrosinase assay was a spectrophotometric analysis based on the methods previously described [33,34,35]. l-DOPA (3,4-dihydroxyphenylalanine) and L-tyrosine were using as the substrate, respectively. In the 96-well microplates, 40 µL of specimen solution and 70 µL of potassium phosphate buffer (0.1 M, pH 6.8) were mixed, which was followed by adding 50 µL of 200 unit/mL mushroom tyrosinase (EC1.14.18.1) after incubation at 25 °C for 10 min. Then, we added 40 µL of 2.5 mM substrate (l-tyrosine/l-DOPA) into the well, and the mixture was incubated for 10 min. After incubation, the absorbance at 475 nm of each well was measured by the ELISA (enzyme-linked immunosorbent assay) reader (SPECTROstar Nano, BMG LABTECH, Offenburg, Germany). The positive control was arbutin, and the number of replications was three. The percentage inhibition of the tyrosinase activity was calculated by the following equation: Inhibition (%) = [(A_control_–A_control’s blank_)–(A_sample_–A_sample’s blank_)/(A_control_–A_control’s blank_)] × 100. Then, the half-maximal inhibitory concentration (IC_50_) of the specimen was calculated from the concentration response curve.

Enzyme kinetic study is analyzed by the Lineweaver–Burk reciprocal plot of the reaction rate and the concentration of the substrate to evaluate the effect of the specimen on the affinity of the substrate and enzyme. The concentration of tyrosinase was kept constant at 200 unit/mL, while substrate (l-tyrosine/l-DOPA) concentration was varied at 0.5, 0.75, 1.0, 1.25, and 1.5 mM. The reaction was similar to antityrosinase assay as described above; 40 µL of specimen solution and 70 µL of potassium phosphate buffer (0.1 M, pH 6.8) were mixed, which was followed by adding 50 µL of 200 unit/mL tyrosinase, after incubation at 25 °C for 10 min. Then, we added 40 µL of substrate into the well and mixed thoroughly, and kinetic measurements of the solution were measured immediately for a period of 3 min at the detected wavelength (475 nm). Both kinetic parameters, Michaelis–Menten constant (K_m_) and maximum velocity (V_max_), were calculated from the Lineweaver–Burk linear equation [36,37,38].

### 2.7. Evaluation of Antimelanogenesis Effect in Zebrafish

Zebrafish (*Danio rerio*) of wild-type AB strain were maintained in a healthy aquatic environment at 26–30 °C, with a 14:10 h light–dark cycle. After 9 h post fertilization (hpf), embryos were placed into a 24-well plate (3 embryos per well), treated with various final concentrations of specimens dissolved in 1% DMSO, and incubated at 28 °C for 48 h (at 57 hpf). The digital image of live zebrafish embryo was taken through a stereomicroscope (Olympus SZ61, Tokyo, Japan) at a total magnification of 40×; then, the melanin content of zebrafish embryo was analyzed by the ImageJ software. Positive controls were arbutin (commercial skin whitening ingredient) and 1-phenyl-2-thiourea (PTU, a melanin synthesis inhibitor). The number of replications was six [21,39,40].

### 2.8. Statistical Analysis

Data obtained in the study were analyzed by Statistical Analysis System (SAS) v 9.2 (Cary, NC, USA) with the Scheffe’s test, which is a post hoc multiple comparison method. The confidence interval was set at 95%.

## 3. Results and Discussion

### 3.1. Antityrosinase Activity of E. serratus Leaf Extract and Its Fractions

**Figure 1:**
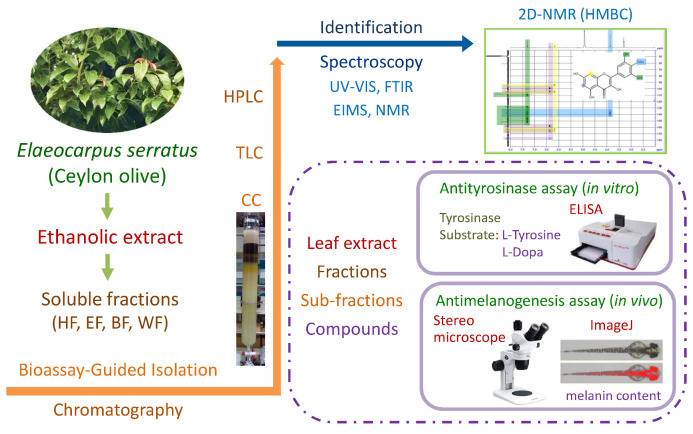
Schematic diagram of antimelanogenesis effects of *E. serratus* leaf extract.

The schematic diagram of extraction, isolation, identification, antityrosinase activity, and antimelanogenesis effect of *E. serratus* leaf extract is shown in Figure 1. The leaf extract was furtherly fractionated into *n*-hexane soluble fraction (HF), ethyl acetate soluble fraction (EF), *n*-butanol soluble fraction (BF), and water soluble fraction (WF) by liquid–liquid partition.

**Figure 2:**
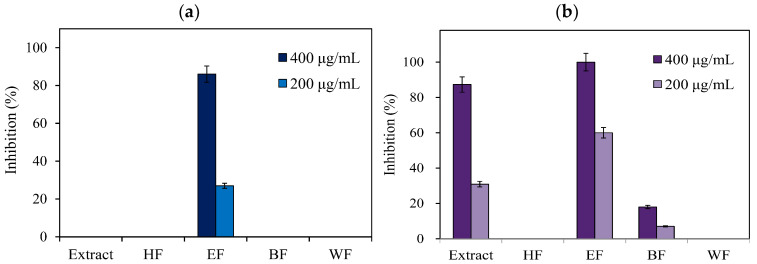
Antityrosinase activities of leaf extract and fractions. (**a**) **l**-Tyrosine as the substrate; (**b**) **l**-DOPA as the substrate.

Tyrosinase, produced by melanocyte cells, plays a key role to catalyze the complicated melanin synthesis; the exploration of tyrosinase inhibitor is one of the prominent ways to retard the melanogenesis [2,5,41]. Tyrosinase inhibition activities of leaf extract and four fractions are shown in Figure 2. When using l-tyrosine as the substrate, only the ethyl acetate soluble fraction had the inhibition effect against tyrosinase at the concentration of 400 µg/mL. Leaf extract, ethyl acetate soluble fraction, and *n*-butanol soluble fraction exhibited the antityrosinase activity when changing l-DOPA as the substrate. Among leaf extract and four fractions, the ethyl acetate soluble fraction demonstrated the best antityrosinase activity with the IC_50_ values of 279.38 and 166.95 µg/mL when using l-tyrosine and l-DOPA as the substrate, respectively (Table 1).

The ethyl acetate soluble fraction was subjected to the bioassay-guided fractionation using preparative column chromatographic and thin-layer chromatographic techniques, and nineteen subfractions (E1–E19) were obtained. Among these subfractions, E7–E10 subfractions showed the higher tyrosinase inhibitory effect in both substrate assays (l-tyrosine and l-DOPA). The half-maximal inhibitory concentration (IC_50_) values of active subfractions against tyrosinase are given in Table 1. The IC_50_ values of all E7–E10 subfractions were below 200 µg/mL; E7 and E9 subfractions even showed better performance compared with arbutin, which is a commercial whitening ingredient.

### 3.2. Isolation and Identification of Compounds from Bioactive Subfractions

**Figure 3:**
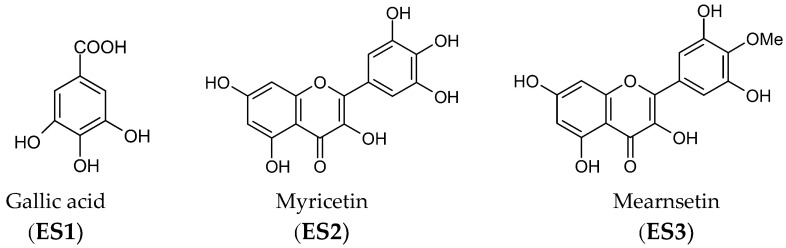
Chemical structures of isolated compounds.

Three compounds (ES**1–3**) were isolated from bioactive subfractions (E7–E10) by high-performance liquid chromatography. Gallic acid (ES**1**): white powder; mp 257 °C; UV (MeOH) λ_max_ (log ε) 215.5 (3.94), 268.0 (3.46) nm; IR (KBr) ν_max_ 3495, 3416, 3285, 1647, 1542, and 1220 cm^−1^; EI-MS m/z 171 [M+H]^+^, in agreement with the molecular formula C_7_H_6_O_5_. Myricetin (ES**2**): yellow needle; mp 358 °C; UV (MeOH) λ_max_ (log ε) 252.5 (4.43) and 374.0 (4.50) nm; IR (KBr) ν_max_ 3421, 1663, 1596, 1520, 1229, 1202, and 1171 cm^−1^; EI-MS m/z 319.36 [M+H]^+^, molecular formula C_15_H_10_O_8_. Mearnsetin (4′-*O*-methyl myricetin, ES**3**): yellow powder; mp 184 °C; UV (MeOH) λ_max_ (log ε) 259.5 (4.21) and 364.5 (4.26) nm; IR (KBr) ν_max_ 3409, 2960, 2927, 2853, 1661, 1599, 1507, 1208, and 1163 cm^−1^; EI-MS m/z 333.0 [M+H]^+^, molecular formula C_16_H_12_O_8_. The NMR data of these compounds are summarized in Table 2. The chemical structures of the identified compounds are shown in Figure 3.

Among these compounds, gallic acid is a phenolic acid; myricetin and mearnsetin belong to the flavonoids. The contents of each compound in ethyl acetate soluble fraction and E7–E10 subfractions are listed in Table 3. The E7 subfraction was rich in mearnsetin (433.38 mg/g); the E8 subfraction primary contained gallic acid (417.64 mg/g). The major constituent of subfractions E9 and E10 was myricetin (406.41 and 336.41 mg/g, respectively). The contents of three compounds in the ethyl acetate soluble fraction were 59.72 mg/g (gallic acid), 45.21 mg/g (myricetin), and 22.66 mg/g (mearnsetin).

### 3.3. Antityrosinase Activity and Enzyme Kinetic Study of Isolated Compounds

Antityrosinase activity of these compounds from active subfractions were represented in Table 4. When using the L-tyrosine as the substrate, the order of antityrosinase activity of examined compounds was mearnsetin > myricetin > gallic acid > arbutin; mearnsetin had the best inhibition effect with the IC_50_ value of 56.57 μg/mL (0.17 mM). Similar results were observed in the l-DOPA used as the substrate; the efficacy of three phytochemicals was superior to that of arbutin.

A true enzyme inhibitor contains four modes of inhibition, including competitive, uncompetitive, mixed type (both competitive and uncompetitive), and noncompetitive [12]. Kinetic constants, K_m_ and V_max_, are determined by the enzyme initial rate at the different substrate concentrations in the Lineweaver–Burk plot; the intersect at the *y*-axis is equivalent to 1/V_max_, and the intersect at the *x*-axis is −1/K_m_. In the enzyme kinetic study of terpenoid compounds from the citrus peel essential oil against tyrosinase, citral acted as a noncompetitive inhibitor, and myrcene was a competitive inhibitor [36]. The inhibition mechanism of 3,7-dioleylquercetin against mushroom tyrosinase was through the competitive inhibition model to suppress the production of melanin [42].

**Figure 4:**
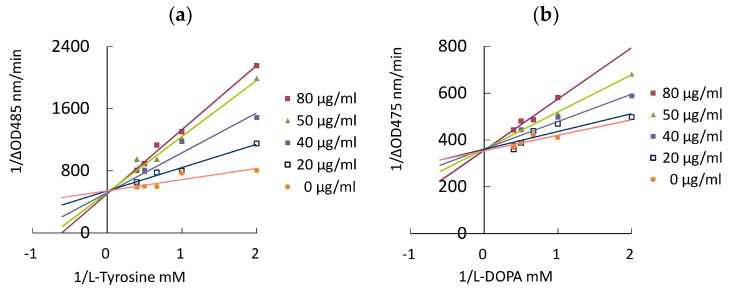
Lineweaver–Burk plots of mearnsetin. (**a**) **l**-Tyrosine as the substrate; (**b**) **l**-DOPA as the substrate.

The inhibition type on tyrosinase of three phytochemicals was elucidated by an in vitro enzyme kinetic study. Figure 4 showed the Lineweaver–Burk plot (double reciprocal plot) of mearnsetin. In the analyses of both substrates, **l**-tyrosine and **l**-Dopa, the linear regression line of different concentrations of mearnsetin had the same intercept on the *y*-axis and the increasing slope. The kinetic parameters of gallic acid, myricetin, and mearnsetin are summarized in Table 5. In the presence of gallic acid or mearnsetin, an increase in K_m_ and a constant in V_max_ were observed, indicating that both gallic acid and mearnsetin were the competitive inhibitor of tyrosinase. It indicated that gallic acid and mearnsetin could bind to free tyrosinase with high affinity and prevent substrate (**l**-tyrosine or **l**-DOPA) binding to the active site of tyrosinase. Myricetin was found to be a mixed-type inhibitor, containing competitive and uncompetitive inhibition, since K_m_ was increased and V_max_ was decreased. Uncompetitive inhibition demonstrated that compounds bind to the tyrosinase–substrate complex but not to the free tyrosinase.

### 3.4. Antimelanogenesis Effects of Extract, Fractions, and Isolated Phytochemicals

**Figure 5:**
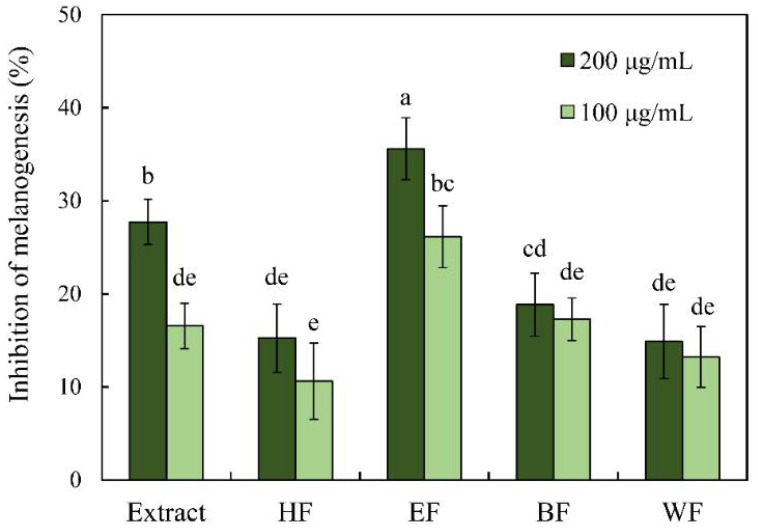
Inhibitory effect of leaf extract and its fractions on the melanogenesis of zebrafish embryo. Different letters (a–e) represent significantly different at the level of *p* < 0.05 according to Scheffe’s test.

Zebrafish (*Danio rerio*) is a novel and valid model organism for melanogenesis research in recent studies [18,19,20,21,40]. The antimelanogenesis effects of leaf extract and four fractions in the in vivo zebrafish assay are shown in Figure 5. The leaf extract and ethyl acetate soluble fraction exhibited good performance in the inhibition of melanin formation in zebrafish embryos. At a concentration of 200 μg/mL, the leaf extract and ethyl acetate soluble fraction inhibited 27.72% and 35.60% of melanin production of zebrafish embryos, respectively. All treatments did not influence the growth of zebrafish embryo with a survival rate of 100%.

**Figure 6:**
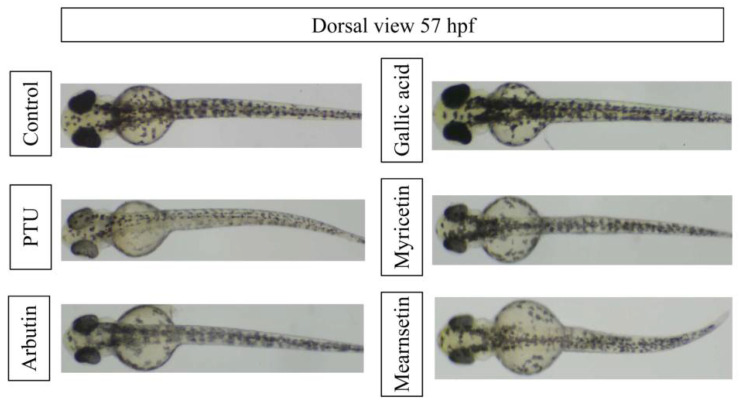
Effects of compounds on melanogenesis of zebrafish at a concentration of 50 μM.

Figure 6 presented the effects of compounds and positive controls, PTU and arbutin, on the melanogenesis of zebrafish at a concentration of 50 μM. PTU is a strong tyrosinase inhibitor to prevent the melanin production, while arbutin is a commercial skin-whitening agent; both positive controls could reduce the melanin formation in zebrafish. Among three phytochemicals, myricetin showed the slight melanin inhibition effect, while mearnsetin had the better melanogenesis inhibition activity in zebrafish.

The effective concentrations of melanogenesis inhibition activity in zebrafish of compounds are listed in Table 6. IC_50_ values of PTU and arbutin were 26.29 μM and 323.69 μM, respectively. The order of antimelanogenesis activity of examined compounds was PTU > mearnsetin > arbutin and myricetin > gallic acid. Mearnsetin exhibited the best efficacy with an IC50 value of 121.01 μM among examined phytochemicals.

## 4. Conclusions

The melanogenesis inhibition effect and antityrosinase activity of leaf extract from Ceylon olive (E. serratus) were evaluated in this study. Among leaf extract and its four fractions, ethyl acetate soluble fraction had the best antityrosinase activity. Phytochemicals, including gallic acid, myricetin and mearnsetin, were isolated and identified from the active subfractions by bioassay-guided chromatography and spectral analyses. The order of antityrosinase activity of these compounds was mearnsetin > myricetin > gallic acid. The in vitro enzyme kinetic study reveals that gallic acid and mearnsetin were competitive inhibitors of tyrosinase, and myricetin was a mixed-type inhibitor. Results from the zebrafish in vivo assay revealed that an ethyl acetate soluble fraction and three phytochemicals possessed significant melanogenesis inhibition effects. The antimelanogenesis effect of mearnsetin was superior to that of the positive control, arbutin, in zebrafish.

## Figures and Tables

**Table 1:** IC_50_ values of active subfractions against mushroom tyrosinase.

Specimen	IC_50_ (μg/mL)	
	l-Tyrosine as the Substrate	l-DOPA as the Substrate
Extract	– *	267.87 ± 2.79 ^B^
EF	279.38 ± 3.06 ^a^	166.95 ± 2.38 ^C^
E7	92.45 ± 2.06 ^d^	126.00 ± 3.43 ^D^
E8	153.97 ± 4.51 ^b^	130.77 ± 0.80 ^D^
E9	125.13 ± 5.01 ^c^	157.05 ± 1.20 ^C^
E10	172.30 ± 2.53 ^b^	180.64 ± 6.61 ^C^
Arbutin **	147.94 ± 1.64 ^b^	305.58 ± 1.28 ^A^

*: > 400 μg/mL; **: Positive control; Different letters (a–d; A–D) represent significantly different at the level of *p* < 0.05 according to Scheffe’s test.

**Table 2:** ^1^H, ^13^C and HMBC NMR data of compounds.

	ES1		ES2			ES3		
Position	^13^C	^1^H	^13^C	^1^H	HMBC	^13^C	^1^H	HMBC
1	122.0							
2	110.6	7.07 (s)	148.0			147.0		
3	146.4		136.9			138.1		
4	139.6		177.3			177.5		
5	146.4		162.5			104.5		
6	110.6	7.07 (s)	99.2	6.17 (d, *J* = 1.95 Hz)	C-5, C-7, C-8, C-10	162.5	6.17 (d, *J* = 1.80 Hz)	C-5, C-7, C-8, C-10
7	170.4		165.6			99.4		
8			94.4	6.37 (d, *J* = 1.95 Hz)	C-6, C-7, C-9, C-10	166.2	6.37 (d, *J* = 1.80 Hz)	C-6, C-7, C-9, C-10
9			158.2			94.5		
10			104.5			158.3		
1′			123.1			128.0		
2′/6′			108.5	7.33 (s)	C-2, C-1′, C-3′/C-5′, C-4′	108.6	7.30(s)	C-2, C-1′, C-3′/C-5′, C-4′
3′/5′			146.7			151.7		
4′			137.4			138.5		
7′						60.8	3.87(s)	C-4′

**Table 3:** Contents of three compounds in the ethyl acetate soluble fraction and active subfractions.

Specimen	Content (mg/g)		
	Gallic Acid	Myricetin	Mearnsetin
EF	59.72 ± 1.08	45.21 ± 0.19	22.66 ± 0.30
E7	- *	-	433.38 ± 3.02
E8	417.64 ± 8.04	132.83 ± 1.56	136.48 ± 3.33
E9	235.04 ± 7.08	406.41 ± 0.41	50.62 ± 1.94
E10	-	336.41 ± 12.12	-

*: Trace or none.

**Table 4:** IC_50_ values of compounds against mushroom tyrosinase.

Specimen	IC_50_	
	l-Tyrosine as the Substrate	l-DOPA as the Substrate
Gallic acid	92.40 ± 4.12 *^,b^ (0.54 ± 0.02) **	106.91 ± 2.19 ^C^ (0.63 ± 0.01)
Myricetin	74.20 ± 7.10 ^c^ (0.23 ± 0.02)	190.78 ± 2.09 ^B^ (0.60 ± 0.01)
Mearnsetin	56.57 ± 1.26 ^d^ (0.17 ± 0.01)	118.92 ± 2.91 ^C^ (0.36 ± 0.01)
Arbutin ***	147.94 ± 1.64 ^a^ (0.54 ± 0.01)	305.58 ± 1.28 ^A^ (1.12 ± 0.01)

*: μg/mL; **: mM; ***: Positive control; Different letters (a–d; A–C) represent significantly different at the level of *p* < 0.05 according to Scheffe’s test.

**Table 5:** Kinetic parameters of phytochemicals against tyrosinase in enzyme kinetic study.

Compound	Substrate	Kinetic Parameter	Concentration (μg/mL)					Potential	Inhibition Type
			0	40	50	80	100		
Gallic acid	l-Tyrosine	V_max_	0.0011	0.0012	0.0012	0.0012	0.0011	―	Competitive
		K_m_	0.0484	0.1458	0.3003	0.7299	0.9054	↑	
	l-DOPA	V_max_	0.0035	0.0034	0.0034	0.0034	0.0035	―	Competitive
		K_m_	0.0733	0.1592	0.2788	0.4385	0.9462	↑	
Myricetin	l-Tyrosine	V_max_	0.0012	0.0010	0.0009	0.0008	0.0007	↓	Mixed
		K_m_	0.1415	0.2997	0.3741	0.3813	0.4225	↑	
	l-DOPA	V_max_	0.0035	0.0028	0.0025	0.0023	0.0020	↓	Mixed
		K_m_	0.1422	0.2877	0.3132	0.3920	0.4068	↑	
Mearnsetin	l-Tyrosine	V_max_	0.0018	0.0018	0.0019	0.0019	0.0020	―	Competitive
		K_m_	0.2673	0.5567	0.9953	1.3976	1.6487	↑	
	l-DOPA	V_max_	0.0028	0.0028	0.0028	0.0028	0.0028	―	Competitive
		K_m_	0.1862	0.2114	0.3217	0.4399	0.6136	↑	

**Table 6:** IC_50_ values of melanogenesis inhibition activity in zebrafish of compounds.

Specimen	IC_50_ (μM)
Gallic acid	― **
Myricetin	349.96 ± 42.67 ^a^
Mearnsetin	121.01 ± 0.96 ^b^
PTU ^*^	26.29 ± 4.17 ^c^
Arbutin ^*^	323.69 ± 19.77 ^a^

^*^ Positive control; ** > 400 μM; Different letters (a–c) represent significantly different at the level of *p* < 0.05 according to Scheffe’s test.

## Data Availability

The data are available are available from the corresponding author on reasonable request.
